# Supplementary material for: Clinical and Metabolic Particularities of a Roma Population with Diabetes—Considering Ethnic Disparities in Approaching Healthcare Management
Source: Biomedicines. 2024 Jun 26;12(7):1422. doi: 10.3390/biomedicines12071422 (PMC11273931; doi:10.3390/biomedicines12071422)
Supplement: Supplementary file 1 [file biomedicines-12-01422-s001.zip › biomedicines-3034811-supplementary.pdf]

Table S1. Prevalence of the patients' comorbidities according to their ethnicity

Comorbidities	Non-Roma patients (n=350)	Roma patients (n=458)	p-value
**Hypertension**	81.1% (n=287)	67.7% (n=310)	0.0001
**Dyslipidemia**	78.5% (n=275)	76.7% (n=351)	0.0001
**Obesity**	50.3% (n=176)	62.2% (n=285)	0.003
**Metabolic syndrome**	89.1% (n=312)	94.3% (n=432)	0.008
**Hepatic steatosis**	55.3% (n=83)	48.5% (n=214)	0.150
**Myocardial infarction**	12.6% (n=44)	12.0% (n=55)	0.809
**Stroke**	5.7% (n=20)	9.2% (n=42)	0.067
**Stable angina**	13.4% (n=47)	29.5% (n=135)	0.0001
**Heart failure**	5.1% (n=18)	19.0% (n=87)	0.0001
**Peripheral artery disease**	21.7% (n=76)	9.6% (n=44)	0.0001
**Lower limb amputation**	3.4% (n=12)	3.5% (n=16)	0.960

Table S2. Prevalence of diabetic complications according to ethnicity

Diabetic complications	Non-Roma patients (n=350)	Roma patients (n=458)	p-value
**Diabetic chronic kidney disease**	34.9% (n=122)	22.1% (n=101)	0.0001
**Diabetic peripheral polyneuropathy**	78.9% (n=276)	72.7% (n=333)	0.044
**Orthostatic hypotension**	9.4% (n=33)	14.6% (n=67)	0.026
**Diabetic retinopathy**	38.3% (n=134)	33.2% (n=152)	0.133

Table S3. Mean values of the analysed parameters according to ethnicity and type of diabetes

Parameters	Non-Roma patients (n=350)		Roma patients (n=458)		p-value**	p-value***
	T1DM (n=17)	T2DM (n=333)	T1DM (n=56)	T2DM (n=402)		
	Mean±SD	Mean±SD	Mean±SD	Mean±SD		
Age (years)	53.65±13.30	62.54±10.25	44.29±13.91	57.24±10.23	<0.001	<0.001
Duration of diabetes (years)	26.00±28.00*	12.00±11.00*	12.00±18.00*	6.00±9.00*	<0.001	<0.001
Height (cm)	163.47±9.40	166.99±9.69	164.74±12.08	164.56±8.77	0.299	0.290
Weight (kg)	69.65±13.24	85.63±17.70	64.49±16.07	91.02±18.26	<0.001	<0.001
WC (cm)	96.98±12.11	105.36±11.76	95.62±13.30	110.58±10.63	0.015	<0.001
HC (cm)	95.00±12.00	104.90±14.09	91.53±8.56	110.96±13.43	0.782	<0.001
BMI (kg/m^2)	32.24±6.00	32.08±6.00	26.12±6.56	33.33±5.99	0.323	<0.001
HbA1c (%)	8.89±1.69	9.06±2.15	10.85±2.28	10.01±2.47	0.369	0.077
FPG (mg/dl)	135.00±215.50*	220.00±125.50*	293.00±205.00*	229.50±153.75*	0.077	<0.001

TC (mg/dl)	176.83±44.74	193.58±66.19	211.00±83.92	217.70±60.57	0.511	0.661
HDL-c (mg/dl)	58.73±16.31	48.92±12.78	48.83±9.77	45.39±8.96	0.017	0.022
TG (mg/dl)	120.00±157.55*	152.25±127.68*	177.36±95.51	214.54±117.00*	0.555	<0.001
LDL-c (mg/dl)	106.00±48.00	102.36±40.90	112.20±39.58	114.09±37.47	0.957	0.956
TyG index	9.15±1.20	9.47±0.78	9.86±0.81	10.10±0.69	0.013	0.075
Creatinine (mg/dl)	0.94±0.28	0.96±0.37	1.12±0.46	1.03±0.43	0.690	0.354
eGFR (ml/min/1.73m²)	82.00±0.10*	82.10±0.11*	82.10±20.00*	81.18±0.10*	0.014	0.43
Urea (mg/dl)	47.77±20.00	44.39±19.99	47.69±25.51	44.02±17.58	<0.001	0.513
Uric acid (mg/dl)	3.50±0.11	5.99±1.98	5.58±2.25	6.26±2.38	<0.001	0.386
UACR (mg/g)	79.57±120.06*	56.50±120.04*	133.07±96.32*	105.00±95.07*	0.676	<0.001
AST (UI/l)	23.00±19.50*	20.00±11.07*	21.00±10.50*	24.00±14.50*	0.627	0.626
ALT (UI/l)	28.00±22.00*	24.00±17.00*	23.50±22.50*	30.00±26.00*	0.727	0.120
GGT (UI/l)	33.35±10.29*	72.83±57.95*	40.00±32.00*	45.00±30.75*	0.373	0.797

Abbreviations: WC (cm)- waist circumference, HC (cm)- hip circumference, BMI (kg/m²)- body mass index, HbA1c (%)- glycated hemoglobin, FPG (mg/dl)- fasting plasmatic glycemia, TC (mg/dl)- total cholesterol, HDL-c (mg/dl)- high-density lipoprotein-cholesterol, LDL-c (mg/dl)- low-density lipoprotein-cholesterol, TG (mg/dl)- triglycerides, TyG index- triglyceride-glucose index, eGFR (ml/min/1.73m²)- estimated glomerular filtration rate, UACR (mg/g)- urinary albumin to creatinine ratio, AST (UI/l)- aspartate aminotransferase, ALT (UI/l)- alanine aminotransferase, GGT (UI/l)- gamma-glutamyl transferase

** between non-Roma and Roma patients with T1DM

*** between non-Roma and Roma patients with T2DM

The data has been represented as mean±SD (standard deviation) and median±IQR (marked with "*", IQR- interquartile range). The statistical significance was considered at a p-value<0.05.

Table S4. TyG index quartiles according to ethnicity

TyG index quartiles	Non-Roma patients (n=350)	Roma patients (n=458)	Total (n=808)	p-value
Quartile 1	34% (n=119)	14% (n=64)	22.6% (n=183)	<0.001
Quartile 2	25.4% (n=89)	36.9% (n=169)	31.9% (n=258)	<0.001
Quartile 3	22% (n=77)	24.7% (n= 113)	23.5% (n=190)	<0.001
Quartile 4	18.6% (n=65)	24.5% (n=112)	21.9% (n=177)	<0.001

TyG index quartiles: quartile 1: <9.36, quartile 2: 9.37-9.91, quartile 3: 9.92-10.45, quartile 4: >10.46

The statistical significance was considered at a p-value<0.05.

Table S5. TyG index quartiles according to ethnicity and type of diabetes

TyG index quartiles	Non-Roma patients (n=350)		Roma patients (n=458)		p-value*	p-value**
	T1DM (n=17)	T2DM (n=333)	T1DM (n=56)	T2DM (n=402)		
Quartile 1	7.6% (n=9)	92.4% (n=110)	15.6% (n=10)	84.4% (n=54)	0.007	<0.001
Quartile 2	2.2% (n=2)	97.8% (n=87)	18.3% (n=31)	81.7% (n=138)	0.005	<0.001
Quartile 3	2.6% (n=2)	97.4% (n=75)	3.5% (n= 4)	96.5% (n=109)	<0.001	<0.001
Quartile 4	6.2% (n=4)	93.8% (n=61)	9.8% (n=11)	89.3% (n=100)	<0.001	<0.001

Abbreviations: T1DM- type 1 diabetes mellitus, T2DM- type 2 diabetes mellitus
TyG index quartiles: quartile 1: <9.36, quartile 2: 9.37-9.91, quartile 3: 9.92-10.45, quartile 4: >10.46
* between non-Roma and Roma patients with T1DM
**between non-Roma and Roma patients with T2DM
The statistical significance was considered at a p-value<0.05.

Table S6. Univariate analysis between TyG index and different variables according to ethnicity

Variables	Coefficient (r)	
	Non-Roma group	Roma group
Age (years)	0.031	-0.052
Duration of diabetes (years)	0.014	-0.047
Weight (kg)	0.118*	0.079
BMI (kg/m^2)	0.094	0.062
WC (cm)	0.116	0.070
HC (cm)	0.033	-0.048
HbA1c (%)	0.473**	0.492**
TC (mg/dl)	0.482**	0.407**
HDL-c (mg/dl)	-0.203**	-0.236**
LDL-c (mg/dl)	0.216**	0.212**
TG (mg/dl)	0.807**	0.792**
Creatinine (mg/dl)	0.091	0.119**
eGFR (ml/min/1.73m^2)	-0.095	-0.075
UACR (mg/g)	0.189**	0.176*
AST (UI/l)	0.057	-0.040

Abbreviations: BMI (kg/m^2)- body mass index, WC (cm)- waist circumference, HC (cm)- hip circumference, HbA1c (%)- glycated hemoglobin, TC (mg/dl)- total cholesterol, HDL-c (mg/dl)- high-density lipoprotein-cholesterol, LDL-c (mg/dl)- low-density lipoprotein-cholesterol, TG (mg/dl)- triglycerides, eGFR (ml/min/1.73m^2)- estimated glomerular filtration rate, UACR (mg/g)- urinary albumin to creatinine ratio, AST (UI/l)- aspartate aminotransferase. *p-value<0.05; ** p-value<0.001
